# Ethical and legislative advances in xenotransplantation for clinical translation: focusing on cardiac, kidney and islet cell xenotransplantation

**DOI:** 10.3389/fimmu.2024.1355609

**Published:** 2024-02-07

**Authors:** Wayne J. Hawthorne

**Affiliations:** ^1^ The Centre for Transplant & Renal Research, Westmead Institute for Medical Research, Westmead, NSW, Australia; ^2^ Department of Surgery, School of Medical Sciences, University of Sydney, Westmead Hospital, Westmead, NSW, Australia

**Keywords:** ethics, guidance’s, hyperacute rejection, legislation, xenozoonosis, xenotransplantation

## Abstract

In this state-of-the-art review we detail the journey of xenotransplantation from its infancy, detailing one of the first published cases and the subsequent journey the field took in its inception and development. With a focus on the science, technological advances, precautions required along with the potential limitations in application, the ethics, guidance’s, and legislative advances that are required to reach the safe and efficacious clinical application of xenotransplantation. Along with a view over the past several decades with the overall significant advancements in pre-clinical study outcomes particularly in islet, kidney, and heart xenotransplantation, to ultimately reach the pinnacle of successful clinical heart and kidney xenotransplants. It outlines the importance for the appropriate guidance’s required to have been developed by experts, scientists, clinicians, and other players who helped develop the field over the past decades. It also touches upon patient advocacy along with perspectives and expectations of patients, along with public opinion and media influence on the understanding and perception of xenotransplantation. It discusses the legislative environment in different jurisdictions which are reviewed in line with current clinical practices. All of which are ultimately based upon the guidance’s developed from a strong long-term collaboration between the International Xenotransplantation Association, the World Health Organisation and The Transplantation Society; each having constantly undertaken consultation and outreach to help develop best practice for clinical xenotransplantation application. These clearly helped forge the legislative frameworks required along with harmonization and standardization of regulations which are detailed here. Also, in relation to the significant advances in the context of initial xeno-kidney trials and the even greater potential for clinical xeno-islet trials to commence we discuss the significant advantages of xenotransplantation and the ultimate benefit to our patients.

## 1 Introduction

**Figure 1:**
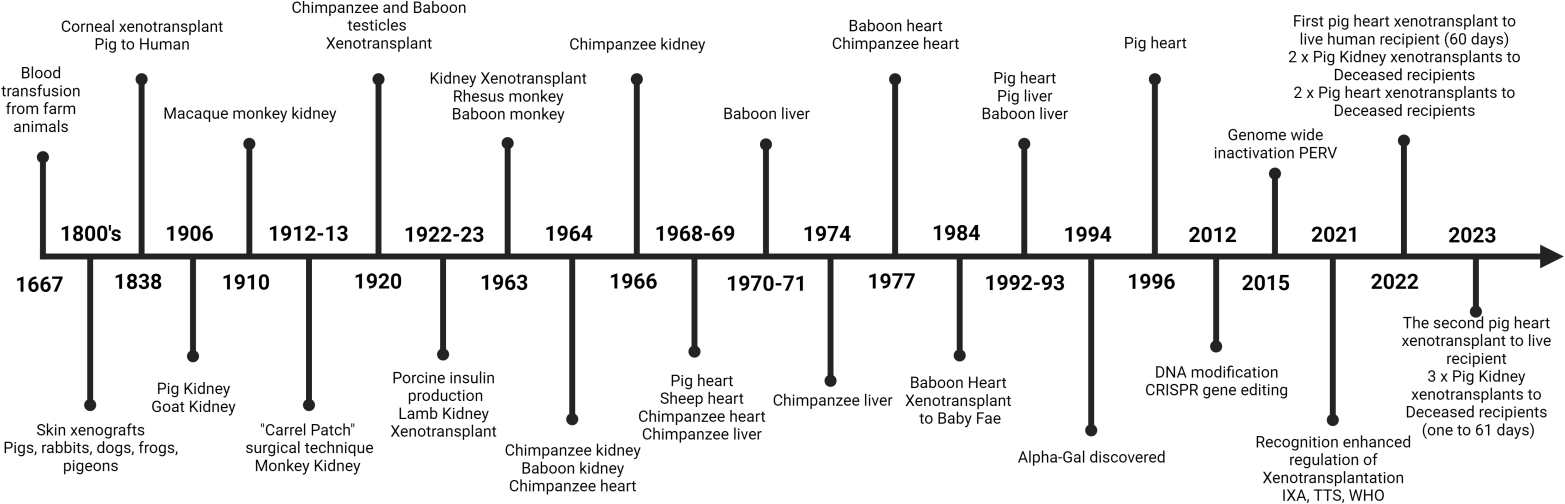
A concise timeline of some of the major developments that have occurred in the field of xenotransplantation. From the first recorded attempt of a xeno-blood transfusion to the current advent of successful clinical xenotransplants of transgenic pig organs to humans.

Xenotransplantation, the latest frontier in transplantation is the process of retrieving organs, tissues or cells from one species and transplanting them into another. It has long been heralded as the ultimate solution to the overwhelming shortage of human organs available for transplantation (1). The concept of utilizing non-human organ and tissue sources to meet the overwhelming demand on conventional donors has captured the attention of clinicians, scientists, healthcare providers, and patients alike for many decades but has also been a concept for hundreds of years (**Figure 1**). As can be seen in **Figure 1**, which is a timeline of some of the major landmarks in the journey of xenotransplantation. The first published attempts of xenotransplantation occurred with xeno-transfusion occurring in the 1600’s then in the 1800’s xeno-skin transplants were attempted prior to more ambitious attempts at kidney xenotransplantation. There has been a long line of endeavor as advancements in medical science and technology have brought the prospect of xenotransplantation closer to reality. Importantly the ethical and legislative landscape surrounding this pioneering field has undertaken renewed and ever-increasing attention but still requires ongoing updates (2–4). A large effort from the International Xenotransplantation Association (IXA) in conjunction with others such as the World Health Organisation (WHO) and the Transplantation Society (TTS) have been constantly undertaken, however as the field progresses more needs to be done from a broader international and national regulatory perspective.

Xenotransplantation offers us the potential to save countless lives by providing a readily available supply of organs, tissues and cells, significantly reducing the waiting time for transplants, and alleviating the suffering of patients on transplant wait lists. It is also a major means by which we can actively abolish the trade in trafficked organs and organ transplant tourism. However, with this promise comes a complex web of ethical considerations and legal frameworks that must be carefully navigated to ensure the responsible and ethical translation of xenotransplantation from the laboratory to the clinic. With this we must ensure that the same endemic issues do not occur with xenotransplantation that have occurred with human organ transplantation such as xenotransplant tourism (5) and unethical processes used to make profits at the expense of the animals used and the patients that may be misled into undertaking unapproved procedures (6).

Historically, xenotransplantation has faced significant challenges, including the perceived/potential for the transmission of diseases from animals to humans (xenozoonosis) (7), concerns over animal welfare (when breeding and producing the donor animals) (8), cultural and religious issues particularly the notion of crossing species boundaries in the use of their tissues for transplantation (6). These challenges led to the imposition of strict regulations along with embargos and a nuanced ethical debate that continues to shape the direction of the field. In recent years, ground-breaking advancements in genetic engineering has rapidly accelerated the field which offers new hope, massively advancing the creation of genetically modified pigs with organs engineered for compatibility with the human immune system (9). These developments have paved the way for the initiation of trials in humans involving xeno-hearts approved under “compassionate use” for live patients (10) along with xeno-hearts and kidneys being studied clinically in a new model using “Brain Dead” recipients (11, 12) along with very successful preclinical trials using transgenic pig islet cells (13).

In this review, we explore the ethical and legislative advances that are underpinning xenotransplantation as it moves toward broadly accepted clinical translation. We delve into the ethical considerations surrounding xenotransplantation, examining questions related to the potential risks of xenozoonotic disease transmission, animal rights, their use, and the public’s perception of this innovative medical approach. We also survey the changes in legislative frameworks governing xenotransplantation, charting their evolution over time, and highlighting the necessity of harmonization and standardisation in regulations worldwide. With a focus on what has been undertaken from the peak governing bodies of the WHO, TTS and the IXA (4)

As the initial clinical trials of xeno-kidneys (12) and hearts (14) bring us closer to the long-awaited reality of xenotransplantation, it is imperative to reflect on the ethical and legislative progress that has brought us to this pivotal moment (4). The careful balance between scientific innovation, human health, and ethical responsibility is at the heart of this transformational journey, and it is through a comprehensive understanding of these advances that we can move forward confidently, ethically and legislatively with the world focusing on xenotransplantation (6).

## 2 Historical perspective

Xenotransplantation has long been heralded as a potential solution to the overwhelming shortage of human organs, tissues and cells available for transplantation (1). The concept of utilizing non-human sources to meet the organ demand has captured the imagination of scientists, healthcare providers, and patients alike. As advancements in science and technology have brought the prospect of xenotransplantation closer to reality, the ethical and legislative landscape surrounding this pioneering field has gained increasing attention especially with the last few years of accelerated progress and commencement of limited life-saving heart and kidney xenotransplantation which have been approved under special compassionate use authorization (i.e., a specific treatment for patients with immediate life-threatening conditions to have access to investigational products outside of an U.S Food and Drug Administration (FDA) -approved clinical trial when no comparable or alternative therapeutic treatment exists to treat the patient’s life threatening illness) (10–12, 15).

Historically, xenotransplantation’s journey has been marked by both hope and challenge. As seen in **Figure 1**. Xenotransplantation has been attempted in many and various settings with many unusual attempts from rather bizarre initial concepts and treatments to now becoming clinical reality. The first published attempt of xenotransplantation took place in the early 17th century when xeno-transfusion was first attempted in June of 1667, in Paris. Jean-Baptiste Denis, a French physician, doctor of King Louis XIV, and Paul Emmerez, surgeon, transfused what we assume to be a small amount of blood from a lamb into a 15-yr-old boy (16). Unfortunately, on the second attempted use of xeno-transfusion it proved unsuccessful and resulted in the death of the patient after which xeno-transfusion was outlawed by the French government (16).

In 1906 the first reported successful kidney xenotransplant was carried out by Mathieu Jaboulay after he and Alexis Carrel perfected the technique of vascular anastomosis. Jaboulay used the vascular technique to transplant a pig kidney onto the brachial artery and cephalic vein of a 48-yr-old woman. Immediately and for the first day and a half he saw significant urine output, but on the third day, he was forced to remove the kidney because of vascular thrombosis (17). Sadly, a lack of understanding of immunology, hematology and any of the intricacies of transplantation, let alone the issues of cross-species xenotransplantation prevented any chance of longer-term success. These early attempts were characterized by a lack of ethical and scientific groundwork, and the risks and consequences of such procedures were often not well understood.

The ensuing centuries saw sporadic and largely unsuccessful attempts at xenotransplantation, with frequent instances of graft rejection and infections that further tempered enthusiasm for the field (18–20). Moreover, as medical ethics evolved and animal welfare concerns gained prominence, the scientific community was challenged to grapple with the significant ethical implications of these procedures, especially when it came to the use of animals involved in the pre-clinical trials and as a source of organs, tissues and cells for transplantation into humans (6).

In the latter half of the 20th century, with the advent of organ transplantation and the increasing demand for donor organs, the potential of xenotransplantation was revisited with renewed enthusiasm pushing the field forward. The discovery of alpha Gal as the mechanism responsible for causing hyperacute rejection (HAR) (21) and the concept of utilizing specifically designed genetically modified pigs (9, 13, 22), capable of providing organs less immunogenic to the human immune system, marked a significant turning point in xenotransplantation’s history. These developments paved the way for the initiation of initial clinical trials involving xeno-kidneys and soon to be islet cell xenotransplants.

As we explore the ethical and legislative advances propelling xenotransplantation toward clinical translation, we must acknowledge the lessons of history. The historical backdrop of early, less-informed attempts, coupled with ethical concerns, has played an instrumental role in shaping the ethical and legislative frameworks we see today (23). The careful balance between scientific innovation, human health, and ethical responsibility is at the heart of this transformational journey. It is through an understanding of these historical challenges that we can appreciate the significance of the ethical and legislative advances discussed in this review, as they propel us closer to the long-awaited reality of xenotransplantation that now seems to be underway (11, 14).

## 3 Major ethical considerations

**Figure 2:**
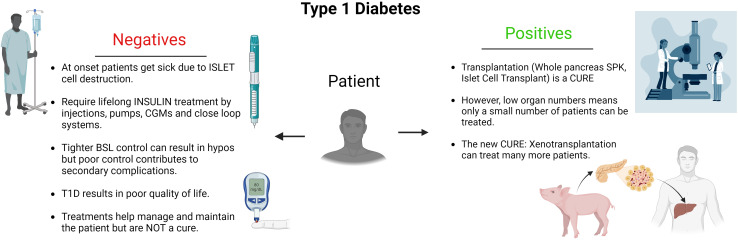
Diagram detailing the conundrum faced by patients suffering from Type 1 diabetes. From the impactful, negative factors affecting them resulting from their disease. To the positive outcomes achieved by having a transplant and the capacity for xenotransplantation to offer more patients a cure.

The remarkable potential of xenotransplantation to address the critical shortage of human organs has been met with considerable ethical scrutiny, raising profound questions and dilemmas that must be thoughtfully addressed. A number of the core areas of ethical concern that have been central to the discourse surrounding xenotransplantation are: the potential for xenozoonosis, public and regulatory issues, crossing of species boundaries and ensuring appropriate animal ethics. However, these must be balanced against the absolute positive gains for the overwhelming number of potential patients that can benefit from xenotransplantation when there are so many medical, financial and social issues for these patients. As can be seen in **Figure 2**, the balance between the negative aspects of their disease versus receiving a cure from the transplant is overwhelmingly weighted to the positive. This is because the benefits far outweigh the problems of ongoing and increasing ill health, secondary complications, invalidity and ultimately death. However, there are not enough human donor organs available for transplantation and using this single example, the case of patients suffering from type 1 diabetes, there are innumerable patients that could benefit from islet cell xenotransplantation with it being life changing and lifesaving.

### 3.1 Potential xenozoonosis

One ethical concern intrinsic to xenotransplantation relates to the potential for the transmission of diseases from animals to humans, a phenomenon known as xenozoonosis. The concept of transmission although theoretical is not unfounded, as various pathogens, including retroviruses, have been identified in pigs could be potential threats in immunocompromised transplant recipients and then theoretically spread to direct close contacts and the broader community (24, 25).

As such this raises potential ethical dilemmas. The duty to protect the broader community and prevent the spread of theoretically potential infectious diseases must be weighed against the need to explore novel medical solutions to help these patients suffering from end stage organ failure and other diseases (6). The possibility of creating animals free from such pathogens as porcine endogenous retroviruses (PERV) through genetic engineering (26) has already been shown to be possible along with raising donor animals in designated pathogen free (DPF) facilities. Along with pigs that have limited pathogens including restricted PERV (27) or where studies have shown no potential for transmission (28, 29). Despite best intensions and even following screening of donor animals we have seen that donor pig organs can still potentially have undetectable porcine viruses such as cytomegalovirus or porcine roseolovirus (PCMV/PRV) detected posttransplant in the donor tissue by plasma microbial cell-free DNA (30). This occurred despite pre-transplant screening and following transplantation into a patient (25).

From a patient and community perspective it is therefore essential for the patients, their family and immediate direct contacts along with the community to understand that if there were in fact a positive case of transmission of a xenozoonosis into a xenotransplant patient that there may well be serious implications to all involved. These implications are potentially as severe as life-long restriction and quarantine of the recipient and may extend to their direct close contacts (25). As part of all Xenotransplantation trial participation it has been advised by the WHO and IXA “Changsha Communique” that all xenograft recipients’ commit to lifelong xenozoonotic monitoring, including agreement to quarantine as a measure to prevent any serious potential spread of infection if detected or suspected (4, 31). As part of the enlistment and education process of patients, patients should be advised of these requirements at the time of prospective trial participation and informed consent process. If the participants choose to they should have the right to withdraw from a xenotransplant trial prior to transplantation. However, once they have been informed, consented and commenced in the trial having undergone xenotransplantation, recipients would be subject to the regulations governing infection containment at a National and International level. Most countries have in place legislation that enforces such quarantinable regulations in relation to communicable diseases (8, 32, 33).

As additional safeguards we also have significant arrays of new antiviral agents capable of eliminating or treating such disease potential (34). Yet, it is essential to ensure that the risk of transmission is minimized and that robust safety measures are in place to protect recipients. This however, does require further address by responsible organizations (WHO, TTS, IXA) and legislators in the many and various international jurisdictions (2–4).

### 3.2 Public and regulatory support

Addressing these ethical concerns is not only a moral imperative but also crucial for gaining public and regulatory support for xenotransplantation. Public perception of the ethics surrounding xenotransplantation can significantly influence its acceptance and, consequently, the regulatory environment. As such there has been significant engagement with both societal and religious organizations to ensure robust understanding of the key concepts and garner opinion and support (6, 35, 36).

The IXA has endeavored to undertake public engagement with ongoing dialogue which are essential to fostering trust with transparency and acceptance. It is incumbent on the broader scientific community and policymakers to communicate the steps taken to mitigate ethical concerns and to provide evidence of the rigorous ethical oversight and animal welfare standards applied in xenotransplantation research. Furthermore the governing organization of xenotransplantation the IXA is maintaining its mission to promote xenotransplantation as a safe, ethical, and effective therapeutic modality by; fostering the science of xenotransplantation through promotion of ethical clinical and pre-clinical research, productive discourse, and collaboration; along with further educating health care providers and lay persons through broad, representative participation in interactive public debate; and also guiding the development of scientifically sound, internationally consistent public policy that is responsive to new developments in the field and acknowledges varying social, ethical and legal frameworks (37). Along with ongoing engagement with regulatory bodies and other agencies to ensure they balance the advancement of science but safeguarding the ethical principles. This is actively being undertaken with a strong push from the IXA to engage numerous agencies globally to ensure this continues to occur and keep pace with the rapidly developing technologies (8).

### 3.3 Crossing species boundaries

Xenotransplantation challenges the traditional conceptual boundaries that separate humans from animals. It poses profound philosophical and ethical questions about the nature of different species and the moral obligation we owe to different species. As we engage in practices that involve genetic modification and the use of animals for human benefit, the ethical boundaries are changing with increasing pressure on ethics committees and legislators to keep track with the pace of change, and we have a moral imperative to ensure that we do keep pace and provide adequate oversight (6).

Some ethicists argue that xenotransplantation exemplifies the Anthropocentric approach (38), emphasizing human interests over those of animals, while others advocate for a more inclusive biocentric perspective that values all forms of life equally (39). The challenge is to find a balance between medical innovation and ethical responsibility to both animal and man (6).

### 3.4 Animal welfare

Xenotransplantation necessitates the use of animals as organ donors. Pigs being primarily used due to their physiological compatibility with humans, their ability to be bred in large numbers at a rapid rate, and their ability to be readily genetically altered. This along with their longstanding acceptance as a source of medical products such as drugs and heart valves and other decellularized tissues. With by far the strongest reason being there acceptance as a major food supply and source of products for man for as long as they have been domesticated should ensure their ease of use ethically (6). However, the welfare of these animals is of paramount concern. As some organizations push the principal that pigs are not merely commodities but sentient beings with the capacity to experience pain and suffering.

The major issues raised are to ensure the donor pigs are being ethically and humanely cared for and ensure the process of genetic manipulation does not cause them any health issues. One could argue that the facilities and conditions that these animals are housed and the care they receive is of a superior level to a normal commercial piggery due to the highly controlled and run facilities including the need for donor animals to be in DPF facilities. Underpinning this is the fact that all animal research projects including the breeding of, care and handling of the animals are undertaken in strict compliance under animal ethics legislation and under scrutiny of ethics committees (6). The genetic modifications required for these donor animals have been carefully designed to ensure they do not affect the health of the source pigs at all. Therefor from an ethical standpoint the level of suffering could be perceived as minimal or negligible. On the other hand, the potential benefit for patients is very high, being lifesaving and life changing (6).

There are also the ethical concerns for pre-clinical study recipients the various animals used and especially the non-human-primates (NHP) which are the benchmark for preclinical trials. Their use is highly recommended prior to acceptance of any program moving to the clinic, and has been advised in many guidance’s such as the “Changsha Communique” that recommend their use to provide safe and efficacious treatment regimen and modalities prior to commencement of any clinical trials (4). So much so that the United States, Food and Drug Administration (FDA) reviewed the NHP preclinical data prior to granting permission for the Baltimore, MD, USA XenoHeart team at the University of Maryland School of Medicine approval for the first pig-to-human heart transplant to be granted (40). The strictest of compliance on ethical grounds is required for any animal study let alone the massive scrutiny undertaken by authorities for NHP research related projects. In most jurisdictions special permission is required, even following appropriate animal ethics approval. Researchers are only allowed to undertake any study with NHPs once accredited and specifically approved due to community concern for their care as they are viewed as so similar to humans.

Scholars and ethicists have explored various strategies to mitigate these concerns. The concept of “minimum moral standing,” as proposed by Rollin, asserts that pigs raised for xenotransplantation should be provided with living conditions and treatment that accord them a minimum level of moral consideration (41). This includes efforts to reduce suffering and enhance the overall welfare of the animals. Ethical guidelines and regulations often inspired by principles of animal welfare, have been developed to ensure humane treatment throughout the animals’ lives and the very best moral and ethical care for all animals.

## 4 Legislative frameworks

Xenotransplantation stands at the intersection of cutting-edge medical science and a complex regulatory landscape. The ethical and safety concerns surrounding xenotransplantation have led to the development of a multifaceted legislative framework designed to ensure both the advancement of this field and the protection of public health.

### 4.1 Existing legislative frameworks

Legislative oversight of xenotransplantation varies across different countries and regions. In the United States (USA), the Food and Drug Administration (FDA) and the Centres for Medicare and Medicaid Services (CMS) (8, 42) are the primary regulatory bodies tasked with overseeing xenotransplantation research and clinical trials. While in China it is the Chinese FDA, Korea (Korean FDA), Argentina (AFDA) whilst in Europe it is the European Medicines Agency (EMA) (43) and in Australia the Therapeutics Goods Administration (TGA) that are charged with establishing regulations and underpinning legislation to support this.

These existing frameworks typically encompass some updates to their regulations, including safety assessments, informed consent, monitoring for xenozoonotic diseases, and research and clinical trial oversight. Such legislation should ideally aim to strike a balance between encouraging scientific innovation and ensuring that risks are rigorously assessed and mitigated with a background based upon preclinical trials, some requiring or suggestive of non-human primate trials as a lead-in to proof of concept for clinical trials (4, 8, 42).

### 4.2 Evolution of legislative frameworks

The legislative landscape for xenotransplantation has evolved significantly over the years. As science has advanced, the regulations have been adapted to keep pace with the changing landscape in transplantation but it still lacks the oversight and ability to completely control all that occurs. Despite the best policies and guidance’s more universal legislation is required to outlaw and prevent organ trafficking and ongoing issues associated with unscrupulous operators (44). The early years of xenotransplantation were characterized by limited regulatory oversight and fragmented approaches to the management of potential risks due to limited legislation to this new field. However, significant oversight was established early on by the WHO, TTS and IXA to ensure there were guidance’s developed to underpin the field (2–4).

However, high-profile setbacks and scientific developments have prompted a revaluation of regulatory frameworks. An example of this was the identification of porcine endogenous retroviruses (PERVs) which raised concerns by government legislators about the potential transmission of these retroviruses to immunocompromised transplant recipients (45). As a result, several jurisdictions-initiated moratorium preventing any clinical xenotransplantation trials from commencing and as such a stronger focus was placed on the assessment and management of this risk in regulatory guidelines (46).

Recently, regulatory bodies have intensified their efforts to provide comprehensive guidelines for xenotransplantation, reflecting a growing recognition of the field’s incredible potential with a balance against the risks. Some guidance’s such as from the USA FDA have addressed issues such as genetic modifications in source animals, monitoring for infectious agents, and the ethical treatment of donor animals (42). And the USA government and other jurisdictions legislating and licensing biological products such as xenografts, tissues and cells under specific biological products legislation (47).

### 4.3 Harmonization and standardization of regulations

One of the most pressing needs in the field of xenotransplantation is the harmonization and standardization of regulations on an international basis. Currently, different countries jurisdictions have distinct legislative frameworks, which can create challenges for researchers and clinicians working in the field. These disparities can hinder the progress of clinical trials and create unnecessary hurdles for advancing this promising technology. Harmonization and standardization are essential for streamlining the path from research to clinical application. By establishing consistent regulations that are internationally recognized and harmonized, xenotransplantation can transcend geographical boundaries, allowing for more efficient and effective collaboration among researchers and acceptance of international clinical trials and also their results (48). As such the IXA in conjunction with the TTS and WHO have for the past decades have been undertaking significant engagement since they combined efforts to establish guidance’s and a xenotransplant registry (49). A significant amount of work has been done by these organizations to ensure there has been expert consultation at an international level. A number of high-level consultations have resulted in the design and development of internationally established guidance’s published under the IXA, TTS, and WHO frameworks with the first published in 2008 with the “Changsha Communique” being drafted and guidance’s now update by multiple panels of international experts on multiple occasions (2–4).

## 5 Clinical xenotransplant studies

The transition from laboratory research to clinical practice is a pivotal phase in the journey of xenotransplantation, and it is marked by the initiation of clinical studies of various kind. These have to date involved the transplantation of organs or tissues from genetically modified pigs into human recipients. In recent years, two types of clinical studies have gained prominence: xeno-heart and kidney transplantation (50–52).

### 5.1 Overview of initial xeno-cardiac, kidney and islet cell trials

#### 5.1.1 Xeno-cardiac and kidney clinical programs

Current clinical studies involving xeno-heart and kidney transplantation have sought to address the critical shortage of available human organs for transplantation. These studies have used specifically developed transgenic pigs that have been genetically modified to be less immunogenic, coagulopathic and prevent hyperacute xenograft rejection.

To date two successful long-term transgenic pig heart xenotransplants into live human patients have been undertaken (40). They have been defined as successful on multiple levels. Firstly, and most importantly they did not undergo hyperacute xenograft rejection, the primary and most significant barrier to xenograft success. Secondly, on the ground of function, these hearts were functional and life supporting for several months. Lastly, the patients were off VA-ECMO, extubated and on no supportive inotropic agents with normal cardiac index and normal biventricular function as demonstrated by echocardiography (14). It is important to understand that both pig-to-human heart xenotransplants were performed following permission for the procedures being granted under Expanded Access authorization by the United Stated, FDA (also known as “compassionate use”) (40).

There have also been several transgenic pig kidney and heart xenotransplants performed in a new clinical recipient research modality. These few early attempts have utilized brain-dead (BD) recipients for transplantation studies and are in their very early stages, having faced various challenges from an ethical perspective. These studies have once again utilized transgenic pigs as the source of donor organs. They have been specifically produced to avoid hyperacute xenograft rejection and provide function in human patients. The first of these kidney studies were taken to only 74 hours posttransplant due to strict ethical constraints. Despite this no hyperacute rejection was observed, and the kidneys remained viable until termination with no chimerism or transmission of porcine retroviruses detected (11). There were two transgenic pig heart xenotransplants also performed in two recently deceased BD recipients. These were only able to be run to 66 hours posttransplant again due to ethical constraints of this model. For both hearts, they also found no evidence of cellular or antibody-mediated rejection, as assessed using histology, flow cytometry and a cytotoxic crossmatch assay. Moreover, they found no evidence of zoonotic transmission from the donor pigs to the human recipients (52).

The transgenic pig kidney xenotransplant studies have continued with several others being undertaken in the same modality in BD recipients. The most recent having been taken out as far as 61 days posttransplant. Despite favorable short-term outcomes and absence of hyperacute injuries, their findings suggest that antibody-mediated rejection in transgenic pig-to-human kidney xenografts might be occurring. The caveat here being the limited transgenesis of these particular donor pig organs playing a significant role (53).

Despite these initial issues and the question of validity of testing the xeno-kidneys in BD recipients due to their altered metabolic state, they represent a promising approach to expand the way to test the safety and efficacy of xeno-organs prior to undertaking xenotransplants in clinical trials. These studies have the potential to increase the data to support the use of xeno-organs to increase the pool of available organs for patients with end-stage renal disease (11, 54).

#### 5.1.2 Islet cell trials

Islet cells, clusters of cells in the pancreas that produce insulin, have been the focus of many pre-clinical trials aiming to provide a treatment for type 1 diabetes. In these studies, islet cells from genetically engineered pigs have been transplanted into various animal models where they have had diabetes induced and are transplanted to potentially restore insulin production. For decades there have also been a significant number of early attempts with both free and encapsulated islets to treat human patients suffering from Type 1 diabetes (55, 56). These have had variable results and no study to date has shown significant change or complete resolution of the recipient’s diabetic state. This has been due to the use in the most part of wild type pig islets rather than purpose developed and bred transgenic pigs (55, 56). However, results from preclinical xeno-islet trials have shown great promise in improving glucose control in non-human-primates establishing it as a potential therapeutic modality for treating diabetic patients (13).

### 5.2 Significance of clinical trials in advancing xenotransplantation

The significance of clinical xeno-heart, kidney and islet cell trials in moving xenotransplantation towards clinical reality cannot be overstated. These trials mark a crucial step in the validation of the safety and efficacy of xenotransplantation in humans. Their outcomes will inform researchers, healthcare providers, regulatory bodies, and the public about the feasibility of this innovative medical approach (57, 58).

Successful trials may also pave the way for wider acceptance of xenotransplantation as a viable solution to the organ shortage crisis. By demonstrating the effectiveness of modified pig organs and addressing safety concerns, clinical trials can build the case for regulatory approval and wider adoption (46, 59).

## 6 Patient perspectives

The success and acceptance of xenotransplantation hinge not only on scientific progress but also on the perspectives and expectations of patients who may ultimately benefit from this innovative medical approach. Understanding the views of prospective recipients and incorporating their voices is essential for the responsible advancement of xenotransplantation (60).

### 6.1 Perspectives and expectations of patients

Patients facing organ failure or debilitating medical conditions have high expectations for xenotransplantation. They see it as a beacon of hope, offering the prospect of a healthier and more fulfilling life. For patients on waiting lists for human organs, xenotransplantation represents a potential lifeline, providing the promise of shorter waiting times and increased access to transplantation.

However, it’s crucial to recognize that patients also have concerns and uncertainties, including the long-term outcomes of xenotransplantation, potential health risks, and the implications of receiving an organ from another species. Patient perspectives encompass a range of emotions, from hope and optimism to apprehension and caution. Addressing these concerns and providing accurate information is paramount in ensuring patient engagement and consent (61).

### 6.2 Informed consent and patient advocacy

In the realm of clinical trials for xenotransplantation, informed consent is a cornerstone of ethical practice. Patients must be fully informed about the experimental nature of the procedure, the potential risks, and the expected benefits. Informed consent allows patients to make autonomous decisions and plays a vital role in respecting their autonomy (39).

Patient advocacy organizations and support networks also play a crucial role in ensuring that patient perspectives are heard and addressed. These organizations work to protect patients’ rights, advocate for transparency, and provide a platform for patients to voice their concerns and expectations. Their role in the xenotransplantation landscape is pivotal in safeguarding the interests of patients.

Patient perspectives and informed consent are not only ethical imperatives but also contribute to the overall success and sustainability of xenotransplantation. By ensuring patients are well-informed and actively engaged in the decision-making process, the field can progress responsibly and ethically, addressing the hopes and concerns of those it aims to benefit (13).

## 7 Public opinion and media influence

Public opinion and media coverage play a pivotal role in shaping the trajectory of xenotransplantation, influencing public perception, regulatory decisions, and the overall direction of this ground-breaking field.

### 7.1 Shaping the future of xenotransplantation

Public opinion wields a considerable impact on the acceptance and progress of xenotransplantation. As a novel medical approach with ethical and scientific complexities, xenotransplantation has the potential to stir both excitement and apprehension among the public. Positive public sentiment can foster support for research, funding, and regulatory approvals, whereas negative perceptions may hinder its advancement (62).

Media coverage significantly influences public opinion by serving as a primary source of information and shaping public discourse. Journalistic narratives can frame xenotransplantation as a ground-breaking medical solution or alternatively in a negative way posing it as a scary and risky endeavor, impacting how it is perceived by the masses (63). It is therefore imperative that the media provides balanced, accurate, and accessible information and in doing so will be vital in shaping the future of xenotransplantation.

### 7.2 Disseminating information and potential misconceptions

Media outlets serve as conduits for disseminating information about xenotransplantation. The media plays an important role in educating the public about the science, ethics, and potential benefits of xenotransplantation. However, the media can also perpetuate misconceptions, oversimplify complex issues, or sensationalize scientific advancements, which may lead to unwarranted public fears and concerns.

The responsible dissemination of information is paramount. Accurate, balanced, and well-informed media coverage is essential in fostering a constructive public dialogue, minimizing misconceptions, and ensuring that public sentiment is based on sound knowledge. Scientists, healthcare providers, and the xenotransplantation community have a shared responsibility to engage with the media to provide accurate and clear information (63, 64).

Public opinion and media influence are pivotal factors in the development of xenotransplantation, influencing the degree of support, funding, and public acceptance. The media’s role in accurately disseminating information and minimizing misconceptions is key to ensuring that public opinion is well-informed and that decisions regarding the future of xenotransplantation are made based on a balanced understanding of the risks and benefits (65).

## 8 International collaboration for xenotransplantation

International collaboration is a cornerstone of xenotransplantation research, and its significance extends to the establishment of common standards, guidelines, and best practices. This global cooperation is crucial for the responsible advancement of the field and the harmonization of regulatory and ethical frameworks.

### 8.1 Importance of global collaboration

Xenotransplantation is not limited by geographic boundaries as seen in the geographical makeup of the broad membership of the IXA and of the significant publications from various units around the world. Researchers, scientists, and healthcare providers contribute their expertise and insights to propel this innovative field forward and the pre-clinical and novel and new use of models such as the BD recipient are synergistic and provide novel information that is perceived to not able to be achieved in NHP. The sharing of knowledge, data, and research findings fosters a collective understanding of the complexities involved in xenotransplantation (66).

Global collaboration is essential in harnessing diverse perspectives and experiences to address common challenges, such as the prevention of zoonotic diseases, the ethical treatment of animals, and the assessment of safety and efficacy (67, 68). This collective effort accelerates the translation of xenotransplantation from research to clinical practice and ensures that there is minimal risk of xenozoonosis or other potential issues (48).

### 8.2 Establishing common standards and guidelines

International collaboration in xenotransplantation research also enables the establishment of common standards, guidelines, and best practices. As the field progresses, consensus on regulatory, ethical, and scientific parameters becomes increasingly vital. Such harmonization streamlines the path from research to clinical application.

Common standards ensure that xenotransplantation research adheres to shared principles, such as animal welfare, patient safety, and ethical practices. International cooperation allows for the identification of gaps and discrepancies in current regulatory frameworks, enabling the development of more comprehensive and universally applicable guidelines such as the “Changsha Communique” (4).

Global collaboration in xenotransplantation research is not merely a choice but a necessity. By pooling resources, knowledge, and expertise from diverse regions, the field can progress with a unified vision. International cooperation helps establish common standards and guidelines, facilitating the responsible and ethical advancement of xenotransplantation and its translation to clinical reality (31) along with ensuring the registries are supported to be able to capture and report on the fields clinical efforts (69).

## 9 Conclusion

The journey of xenotransplantation, the transplantation of organs or tissues from one species to another, has witnessed significant advancements and encountered ethical, legislative, and scientific challenges. This review has delved into various facets of xenotransplantation, emphasizing its potential to address the critical organ shortage crisis while highlighting the essential elements required for its responsible and successful translation to clinical reality.

### 9.1 Take home messages

#### 9.1.1 Ethical and legislative advances

The historical context, ethical considerations, and legislative frameworks have been pivotal in shaping the path of xenotransplantation. From early attempts at cross-species transplantation to the contemporary emphasis on animal welfare and informed consent, the field has evolved significantly.

#### 9.1.2 Advancements in genetic engineering

Genetic engineering has ushered in a new era for xenotransplantation, allowing for the creation of genetically modified pigs with organs more compatible with human recipients. These “designer pigs” represent a breakthrough in reducing immunological barriers.

#### 9.1.3 Clinical xenotransplant trials

The initiation of clinical studies involving xeno-hearts, kidneys and islet cells marks a critical step in validating the safety and efficacy of xenotransplantation in humans. These studies can move forward to trials which hold the potential to significantly expand the pool of available organs and improve treatment options for many diseases and conditions.

#### 9.1.4 Patient perspectives

Patients eagerly anticipate the prospects of xenotransplantation, viewing it as a lifeline for lifesaving or life-improving interventions. Understanding and addressing their perspectives and expectations are essential for responsible clinical progress.

#### 9.1.5 Public opinion and media influence

Public opinion and media coverage play a substantial role in shaping the future of xenotransplantation. The media’s role in disseminating accurate and balanced information is critical in fostering constructive public dialogue and minimizing misconceptions.

### 9.2 Continuing ethical and legislative advancements

Ethical and legislative advancements are indispensable as xenotransplantation moves closer to clinical translation. The responsible treatment of animals, transparent informed consent, and comprehensive regulatory frameworks are fundamental to ensuring ethical and safe practices.

### 9.3 Alleviating the organ shortage crisis

The potential of xenotransplantation to alleviate the organ shortage crisis cannot be overstated. As clinical trials progress and demonstrate the viability of xenotransplantation, it stands as a beacon of hope for those awaiting life-saving organ transplants.

### 9.4 Promising future for xenotransplantation

The promising future for xenotransplantation lies in its potential to bridge the gap between the demand for organs and their limited supply. With continued collaboration, ethical diligence, and advancements in science, xenotransplantation can move from the realm of theoretical possibility to practical reality.

To bring xenotransplantation to the clinic, the scientific community, regulatory bodies, and the media must work in harmony. International collaboration is essential to continue to establish common standards and guidelines, enabling the field to progress responsibly and ethically in a universal fashion on an international stage.

As we navigate the uncharted frontiers of xenotransplantation and further clinical application, ethical decisions and legislation that accompany it, the future looks promising, provided we remain steadfast in our commitment to science, ethics, and the well-being of both humans and animals. This review underscores the remarkable potential of xenotransplantation while recognizing the importance of treading the path to the clinic with care, diligence, empathy, and informed action including harmonization of guidance’s’ and legislation internationally.

## Author contributions

WH: Conceptualization, Writing – original draft, Writing – review & editing.
